# Association of Alcohol Consumption with Specific Biomarkers: A Cross-sectional Study in South Africa

**Published:** 2015-03

**Authors:** Pedro T. Pisa, Hester H. Vorster, Annamarie Kruger, Barrie Margetts, Du T. Loots

**Affiliations:** ^1^Centre of Excellence for Nutrition, North-West University, Potchefstroom 2520, South Africa; ^2^MRC/Wits Developmental Pathways for Health Research Unit, Department of Paediatrics, Faculty of Health Sciences, University of the Witwatersrand, Johannesburg, South Africa; ^3^Institute of Human Nutrition; University of Southampton, Southampton, United Kingdom; ^4^Division of Biochemistry, School for Physical and Chemical Sciences, North-West University, Potchefstroom 2520, South Africa

**Keywords:** Africans, Alcohol consumption, Gamma glutamyl transferase, Percentage carbohydrate-deficient transferrin, PURE study, Transition, South Africa

## Abstract

Alcohol consumption plays an important role in the health transition associated with urbanization in developing countries. Thus, reliable tools for assessing alcohol intake levels are necessary. We compared two biological markers of alcohol consumption and self-reported alcohol intakes in participants from urban and rural South African communities. This cross-sectional epidemiological survey was part of the North West Province, South African leg of the 12-year International Prospective Urban and Rural Epidemiology (PURE) study which investigates the health transition in urban and rural subjects. A total of 2,010 apparently healthy African volunteers (35 years and older) were recruited from a sample of 6,000 randomly-selected households. Alcohol consumption was assessed through self-reports (24-hour recalls and quantitative food frequency questionnaire) and by two biological markers: percentage carbohydrate-deficient transferrin (%CDT) and gamma-glutamyl transferase (GGT). Of the 716 men and 1,192 women volunteers, 64% and 33% respectively reported regular alcohol consumption. Reported mean habitual intakes of drinker men and women were 29.9 (±30.0) and 23.3 (±29.1) g of pure alcohol per day. Reported habitual intake of the whole group correlated positively and significantly with both %CDT (R=0.32; p≤0.01) and GGT (R=0.43; p≤0.01). The correlation between the two biomarkers was low (0.211; p≤0.01). GGT and %CDT values should be interpreted with care in Africans as self-reported non-drinker men and women had elevated levels of GGT (19% and 26%) and %CDT (48% and 38%). A need exists for a more specific biological marker for alcohol consumption in black Africans.

## INTRODUCTION

Due to rapid urbanization, South Africa is experiencing a health transition associated with a triple burden of disease characterized by a high prevalence of undernutrition-related infectious diseases, the emergence of non-communicable diseases, and the human immunodeficiency virus/acquired immune deficiency syndrome (HIV/AIDS) pandemic (1). The World Health Organization (WHO) recently stated that alcohol consumption is the fifth leading cause of death worldwide and that intakes are on the increase, especially in developing countries (2). According to the WHO's database, South Africa's adult per-capita consumption of pure alcohol is 9.46 litre. What is, however, of concern is that those who reported to drink have a 34.90 litre per-capita consumption of pure alcohol by adults for both genders, which is one of the highest in the world (2). Alcohol-abuse in South Africa is reported to be responsible for at least half of the 14,000 annual road deaths, high crime rates, violence, sexual risk behaviour, family disruption, and a host of individual and societal problems (3). Additionally, binge drinking results in an increased cardiovascular disease (CVD) risk as well as micronutrient deficiencies (4) both having high prevalence in the South African population (1).

Epidemiological evidence suggests a J- or U-shaped relationship between alcohol consumption and CVD (5–7). There is a need to assess, with accuracy, the high-risk drinking in this population and to relate alcohol intakes (exposure) to different health outcomes.

Identification and assessment of high-risk drinking in a population can be problematic when self-reports of alcohol consumption is used as an indicator as there is usually under- or overreporting by the respondents. Although a detailed, validated quantitative food frequency questionnaire (QFFQ) is an important source of consumption information (8) and typically has low rates of false-positive responses, the primary weakness is that people struggle to report their alcohol consumption levels accurately (9). Underreporting has been shown to be common in population surveys (10), and this is largely attributed to the low participation by alcoholics and heavy drinkers in these surveys. Additionally, there is the tendency of respondents, especially those that are alcohol-dependent to underreport their consumption in questionnaire and interviews (11,12).

There are biological markers, such as circulating carbohydrate-deficient transferrin (CDT) and gamma glutamyl transferase (GGT) that are sensitive to high alcohol consumption and are suitable biomarkers for identifying alcohol-use or abuse in most populations (13,14). These biomarkers may be used in estimating or verifying reported alcohol consumption or real consumption rates in different populations; %CDT, which measures the relative amount of CDT isoforms in proportion to total transferrin, has been shown to be a slightly better marker compared to absolute CDT values (15–22).

It is important to use these biomarkers to verify reported intakes and to identify and assess high-risk drinking patterns with better accuracy in African populations. The aim of this study was to assess cross-sectionally the self-reported alcohol consumption and its association with the levels of %CDT and GGT in a randomized sample in the North-West Province of South Africa.

## MATERIALS AND METHODS

### Study design and subjects

This cross-sectional epidemiological survey is part of the North-West Province, South African leg of the 12-year PURE study which investigates the health transition in urban and rural subjects. The main selection criteria for recruitment in this study were that (i) there should be migration stability within the chosen rural and urban communities and (ii) participants should be older than 35 years of age, with no reported chronic diseases for lifestyle, tuberculosis, and known HIV infection. The baseline data were collected from October to December 2005. A total of 1,006 rural and 1,004 urban black South Africans were recruited from a sample of 6,000 randomly-selected households. Permission to conduct the study in the abovementioned communities, with advice on recruitment procedures, was obtained from the North-West Department of Health, tribal chiefs, community leaders, employers, and mayors. The study procedure was explained to participants in their home language, after which participants signed informed consent forms, and the study commenced. All data were treated confidentially, and all analyses were performed with coded data.

### Assessment of alcohol consumption

A structured and validated dietary questionnaire QFFQ (8) and a single 24-hour recall (randomly conducted from Monday to Friday) were used in collecting dietary data from each subject by 16 intensively-trained fieldworkers. The dietary data from the two dietary methods were coded by two dieticians and sent to the Medical Research Council of South Africa for computerization, cleaning, and nutrients analyses. The dietary data included responses to two sets of questions regarding alcohol consumption: the quantity and frequency question from the food frequency questionnaire and the daily consumption from the 24-hour recall. In both sets of questions, intakes of different beverages were assessed separately. Average alcohol intake was estimated by the amount of alcohol consumed per day and expressed as intake of pure alcohol in grammes per day. Calculations were based on the South African Food Composition Tables (23).

### Blood pressure, electrocardiogram, and anthropometry measurements

Blood pressure was measured using the Omron automatic digital blood pressure monitor (Omron HEM-757-UK), blood glucose (Vitros DT6011 Chemistry Analyzer, Ortho-Clinical Diagnostics, Rochester, New York, USA), and anthropometric measurements (height, weight, waist- and hip-circumference, mid-upper arm-circumference, triceps skinfold, calf-circumference, calf skinfold, supra-spinal skinfold, upper flexed arm-circumference) were made using the guidelines adopted at the NIH-sponsored Arlie Conference (24). An electrocardiogram lung function test was done using spirometer.

### Biochemical analyses

Registered nurses collected venous blood samples, and participants were asked to fast overnight (8 hours with no food or beverage, excluding water). Fasting blood samples were collected between 08:00 and 11:00 am. For the biochemical analysis of variables reported in this study, serum samples were used and were prepared by collecting blood in tubes without anticoagulant, and blood was left to clot. Samples were centrifuged at 2,000 g for 15 minutes at 10 degrees within 30 minutes after collection. Aliquots were frozen on dry ice, stored in the field at −18 °C and then, after 2-4 days, at −82 °C until analysis. Quantitative determination of GGT, total protein (T-protein), albumin, blood glucose, high-density lipoprotein cholesterol (HDL-C), and iron levels were measured in these serum samples by Sequential Multiple Analyzer Computer (SMAC), using the Konelab^TM^ auto-analyzer (Thermo Fisher Scientific Oy, Vantaa, Finland), a clinical chemistry analyzer for colorimetric, immunoturbidimetric, and ion-selective electrode measurements. Ready-to-use reagents were placed on a cooled reagent disc with 35 positions; the samples calibrators and controls were placed on the samples disc. Pre-dilution of samples and post-dilution with high and low secondary dilution ratios were handled automatically. Calibration curves and controls were applied to ensure quality of the procedure performed. The cutoff values for GGT were set at 80 units per litre (U/L) and 50 U/L for men and women respectively. Serum %CDT analyses were performed by using an *in vitro* heterogeneous immunoassay with column separation, followed by a turbidimetric measurement (Axis-Shield %CDT kit, Oslo, Norway). The measuring range of this test is 1.5 to 24 mg/L, and cutoff values for %CDT were set at 2.6% following recommendations of the manufacturer; %CDT assays use a microcolumn separation procedure after total iron saturation. Sialic-deficient transferrin (asialo, monosialo, and diasialo) are separated from natural transferrin and quantified, and the results are expressed in %CDT (of total transferrin in the sample before column chromatographic separation). A calibration curve was established based on natural (total) transferrin to quantify unknown samples (triplicate high and low controls were run and computed before every run). The coefficient of variance for both intra- and inter-plate assays for %CDT was <10%.

### Statistical methods

Data were analyzed using the SPSS (version 17). Means, medians, standard deviations, and 95% confidence intervals were calculated. As data were not normally distributed, non-parametric tests were used in assessing correlations and differences between groups. Wilcoxon Signed Rank tests and Mann-Whitney/Wilcoxon Rank Sum tests were used for comparing groups. Spearman rank-order and partial correlations were used in examining the associations between self-reported alcohol consumption and biomarkers while the latter method was used for assessing associations after adjustments for confounding factors. Body mass index (BMI) and smoking emerged as valid confounders for this population. Drinkers and non-drinkers and men and women were analyzed separately. Further stratification of alcohol consumption into 4 groups (0, >0.01-15.99, 16.00-30.00, and ≥30.01 g/day of pure alcohol) from the QFFQ was done, based on recommendations that daily alcohol consumption should be approximately 20 g for men and 15 g for women (25,26), and light-to-moderate alcohol intake being estimated to be <30 g, and heavy drinking amounting to intakes of >30 g of absolute alcohol daily (7).

### Ethical approval

The study was approved by the Ethics Committee of the North-West University, Potchefstroom, South Africa (Number: 04M10).

## RESULTS

Table 1 summarizes the baseline characteristics of the participants in the PURE study. Self-reported mean consumption of pure alcohol in g/day by two different dietary methods for the PURE participants is shown in Table 2. The estimated average alcohol intakes from the two dietary methods were significantly different. The values for QFFQ which measures habitual alcohol intake was higher than that for the 24-hour recall for both men and women. Self-reported mean intakes of men were higher than that for women, using both dietary methods. As for self-reported drinkers only, reported mean alcohol consumption from the QFFQ was more than double that as reported using 24-hour recall method for both men and women. Table 3 reports the associations between the biological markers and self-reported intakes. A statistically significant correlation between the two different dietary methods was observed, higher than +0.45 (p≤0.01) in both women and men. In both men and women, GGT had a stronger correlation with reported alcohol consumption measures than %CDT. The same pattern was also shown amongst drinkers only. The correlation between %CDT and GGT was, however, low (0.211, p≤0.01). After controlling for BMI and smoking, all correlations became weaker. The expected relationships of alcohol and biochemical and physiological variables known to be influenced by alcohol were also observed in this population; %CDT, GGT, albumin, iron, and HDL-C were significantly higher in the drinkers in both men and women, and BMI was significantly lower in drinkers (Table 4). Mean %CDT and GGT for each group and percentage subjects with elevated values are shown in Table 5; %CDT and GGT levels were generally higher in men than women. Self-reported non-drinkers showed elevated GGT (18.9 and 25.6% in men and women respectively) and %CDT levels (47.7 and 38.1% in men and women respectively).

**Table 1.** Mean (SD) baseline characteristics of participants in the PURE study

Variable	Men (N=716)	Women (N=1,192)
Age (years)	49.75 (10.30)	49.10 (10.37)
Reported alcohol intake (g/day) (QFFQ)		
Whole population	19.20 (28.00)	7.70 (20.10)
Self-reported drinkers	29.90 (30.00)	23.30 (29.10)
Self-reported non-drinkers	0.00 (0.00)	0.00 (0.00)
Smoking cigarettes/day	4.07 (4.71)	2.61 (3.48)
Body mass index (kg/m^2^)	20.79 (4.04)	26.90 (7.32)
Blood pressure (mmHg)		
Systolic	138.16 (31.34)	134.17 (24.94)
Diastolic	86.67 (14.37)	88.31 (14.22)
Plasma lipids (mmol/L)		
High-density lipoprotein-cholesterol	1.58 (0.66)	1.48 (0.62)
Triglycerides	1.22 (0.86)	1.34 (0.75)
Total cholesterol	4.81 (1.34)	5.13 (1.39)
Gamma-glutamyl transferase (U/L)	127.27 (241.78)	79.58 (163.18)
Percentage carbohydrate-deficient transferrin	3.49 (1.68)	2.64 (1.12)

N=Number

QFFQ=Quantitative food frequency questionnaire

SD=Standard deviation

## DISCUSSION

This is the first study to explore the associations between %CDT and GGT levels with self-reported alcohol intakes from a structured culturally-sensitive validated QFFQ (8) and 24-hour recall methods in an African population where the estimated alcohol consumption per capita is among the highest in the world (3). This study reports high alcohol intakes for both men and women. Both %CDT and GGT had relatively good correlations with reported intakes but self-reported non-drinking men and women had elevated GGT (18.9 and 25.6% in men and women respectively) and %CDT levels (47.7 and 38.1% in men and women respectively). Between the two reporting methods, the QFFQ seems a more accurate method to use in epidemiological alcohol studies as it illustrates the habitual alcohol consumption whereas the 24-hour recall method reports intakes based only on the previous 24 hours (short-term). Although the latter is an open-ended instrument, when not repeated as in this study, it might not fully capture usual intakes of alcohol. In this study, 24-hour recall questionnaire was completed randomly throughout the households from Monday to Friday. The pattern of alcohol consumption in this population tends to be heavy drinking at weekends, making the 24-hour recalls particularly less convincing tools in this study. Because of the fieldwork logistics around data collection in the setting of this study, it was not feasible to administer 24-hour recalls on weekends.

In African settings, including the one under investigation, women may be more prone to underreporting alcohol intakes due to cultural and traditional norms of women being viewed with no respect if known to be alcohol consumers. It has long been hypothesized that woman who consume alcohol are of promiscuous behaviour or lack morals; thus, most woman tend to hide or lie about alcohol intake. Although this type of stigmatization is evolving due to urbanization, most African settings remain traditionally conservative. African men may also overestimate their intakes as drinking is associated with masculinity but, with more public health awareness programmes, underreporting is also possible as people wish to be associated with healthier lifestyle. It should be noted that error would likely be present (under- or overreporting) in the actual intake reported but the ranking would be the same meaning that this won't affect the correlation coefficients. Thus, error remains mostly random, which possibly can weaken association but will not introduce much bias.

**Table 2.** Comparison of mean (SD) of self-reported alcohol consumption by two different methods (24-hour recall and QFFQ) by gender and age-group

Characteristics	Age-groups and mean (SD) age (completed years)	24-hour recall method			QFFQ method			Test statistic^c^
		Mean alcohol intake (SD)			Mean alcohol intake (SD)				
		N	(g/day)	95% CI	N	(g/day)	95% CI	z-score
Total group								
Men	35-44	248	12.0 (29.9)	8.3,15.8	245	20.7 (31.9)	16.7,24.7	-5.194^b^*
	45-54	239	14.5 (30.8)	10.6,18.4	238	19.1 (24.3)	16.0,22.2	-3.808^b^*
	55-64	159	12.3 (26.5)	8.1,16.4	156	19.7 (27.8)	15.3,24.1	-3.729^b^*
	65-74	51	4.0 (14.8)	-0.1,8.2	51	14.4 (26.9)	6.8,22.0	-3.436^b^*
	>75	13	4.3 (11.6)	-2.7,11.3	12	9.7 (20.6)	-3.4,22.7	-0.135^b^ (NS)
Total men	49.8 (10.3)	716	12.2 (28.4)	10.1,14.3	708	19.2 (28.0)	17.0,21.2	-8.000^a^*
Women	35-44	454	3.6 (15.4)	2.1,5.0	450	7.7 (18.6)	6.0,9.5	-6.850^b^*
	45-54	394	3.7 (16.0)	2.1,5.3	388	7.7 (20.1)	5.7,9.7	-6.336^b^*
	55-64	222	4.3 (17.2)	2.0,6.5	220	8.8 (23.8)	5.6,12.0	-4.467^b^*
	65-74	85	0.5 (4.3)	-0.5,1.4	83	6.2 (18.5)	2.2,10.3	-3.825^b^*
	>75	26	0.0 (0.0)	0.0,0.0	26	3.1 (15.7)	-3.2,9.5	-1.342^b^ (NS)
Total women	49.1 (10.4)	1,192	3.4 (15.2)	2.5,4.3	1,178	7.7 (20.1)	6.5,8.9	-11.196^a^*
Self-reported drinkers							
Men	49.5 (9.5)	454	18.4 (33.4)	15,21	454	29.9 (30.0)	27,33	-8.378^b^*
Women	48.0 (9.0)	392	9.9 (25.0)	7,12	391	23.3 (29.1)	20,26	-11.722^b^*

^a^Based on negative ranks

^b^Based on positive ranks

^c^Wilcoxon Signed Ranks Test

*Significant differences (p≤0.05) between 24-hour recall and QFFQ method

CI=Confidence interval

NS=Not significant

SD=Standard deviation

QFFQ=Quantitative food frequency questionnaire

N=Number of subjects

**Table 3.** Correlations between gamma glutamyl transferase (GGT), percentage carbohydrate-deficient transferrin (%CDT), and self-reported alcohol consumption for total sample, stratified by gender, urbanization, and drinking status

Characteristics	%CDT r_s_	GGT r_s_	QFFQ method r_s_	24-hour/recall method r_s_	%CDT R	GGT R
Total sample						
QFFQ method	0.320**	0.433**	1.000	0.472**	0.193#	0.310#
24-hour/recall method	0.205**	0.321**	0.472**	1.000	0.165#	0.264#
%CDT	1.000	0.211**	0.320**	0.205**	1.000	0.110#
GGT	0.211**	1.000	0.433**	0.321**	0.110#	1.000
Gender						
Male						
QFFQ method	0.333**	0.369**	1.000	0.458**	0.197#	0.291#
24-hour/recall method	0.222**	0.310**	0.458**	1.000	0.167#	0.301#
%CDT	1.000	0.253**	0.333**	0.222**	1.000	0.118#
GGT	0.253**	1.000	0.369**	0.310**	0.118#	1.000
Female						
QFFQ method	0.198**	0.398**	1.000	0.411**	0.097#	0.314#
24-hour/recall method	0.088**	0.273**	0.411**	1.000	0.065	0.187#
%CDT	1.000	0.098**	0.198**	0.088**	1.000	0.072
GGT	0.098**	1.000**	0.398**	0.273**	0.072	1.000
Rural-Urban						
Rural						
QFFQ method	0.350**	0.452**	1.000	0.454**	0.250#	0.331#
24-hour/recall method	0.189**	0.300**	0.454**	1.000	0.188#	0.267#
%CDT	1.000	0.209**	0.350**	0.189**	1.000	0.145#
GGT	0.209**	1.000	0.452**	0.300**	0.145#	1.000
Urban						
QFFQ method	0.298**	0.387**	1.000	0.477**	0.088	0.259#
24-hour/recall method	0.220**	0.320**	0.477**	1.000	0.112#	0.241#
%CDT	1.000	0.217**	0.298**	0.220**	1.000	0.033
GGT	0.217**	1.000	0.387**	0.320**	0.033	1.000
Self-reported drinkers						
Drinkers						
QFFQ method	0.148**	0.275**	1.000	0.382	0.068	0.189#
24-hour/recall method	0.135**	0.270**	0.382**	1.000	0.101#	0.194#
%CDT	1.000	0.093*	0.148**	0.135**	1.000	0.009
GGT	0.093*	1.000	0.275**	0.270**	0.009	1.000

**Correlation significant at the p≤0.01 level (2-tailed)

*Correlation significant at the p≤0.05 level (2-tailed)

#Partial correlation significant at the p≤0.05 level (2-tailed)

R=Partial correlation after adjusting for BMI and smoking

r_s_=Spearman correlation coefficient

**Table 4.** Comparison of mean (SD) and median of biochemical, physiological and dietary data of drinkers and non-drinkers stratified by gender

Variable	Men						Women					
	Drinkers			Non-drinkers			Drinkers			Non-drinkers		
	Mean (SD)	Median	95% CI	Mean (SD)	Median	95% CI	Mean (SD)	Median	95% CI	Mean (SD)	Median	95% CI
Age (years)	49.5 (9.5)	48.0	48.6,50.3	50.3 (11.6)	48.0	49.0,52.0	48.0 (9.0)	46.0	47.0,48.9	49.8 (11.0)	48.0	49.0,51.0
BMI (Kg/m^2^)	20.2* (3.6)	19.5	19.9,20.6	21.9 (4.6)	20.9	21.4,23.0	25.4# (7.3)	25.4	24.7,26.2	27.5 (7.2)	26.7	27.0,28.0
Smoking	5.0* (4.9)	4.0	4.5,5.5	2.4 (4.1)	0.0	2.0,3.0	3.5# (3.6)	3.5	3.0,3.9	2.2 (3.4)	0.0	2.0,2.5
T-protein (g/L)	84.2 (15.8)	81.9	82.7,85.7	85.2 (17.5)	81.0	83.0,87.4	85 (17.9)	82.0	84.0,87.7	87.0 (18.2)	83.9	85.6,88.3
Albumin (g/L)	46.3* (13.7)	42.7	45.0,47.6	49.0 (13.0)	44.0	47.4,51.0	46.1# (13.3)	42.6	44.7,47.5	48.5 (12.5)	43.7	47.5,49.3
Serum iron (mmol/L)	23.4* (15.3)	18.7	21.9,24.8	20.5 (14.6)	16.2	18.7,22.4	21.6# (15.7)	17.3	20.0,23.3	16.2 (10.5)	13.8	15.4,17.0
%CDT	3.8* (1.7)	3.4	3.7,4.0	2.9 (1.4)	2.5	2.7,3.1	3.0# (1.3)	2.8	2.9,3.1	2.4 (1.0)	2.3	2.3,2.5
GGT (U/L)	152.5* (211.9)	81.9	132.3,172.8	91.0 (296.5)	43.9	54.0,128.4	131.5# (194.0)	67.1	111.6,151.4	50.7 (65.1)	33.7	46.0,55.4
HDL-C (mmol/L)	1.7* (0.7)	1.7	1.6,1.8	1.4 (0.6)	1.2	1.3,1.4	1.6# (0.7)	1.5	1.5,1.7	1.4 (0.5)	1.3	1.4,1.5
Fasting blood glucose (mmol/L)	5.3 (1.4)	5.2	5.1,5.4	5.4 (1.2)	5.3	5.2,6.0	5.6 (1.7)	5.5	5.3,5.7	5.7 (1.7)	5.4	5.5,5.8

*Significant difference between men drinkers and non-drinkers (Mann-Whitney U-test, p≤0.05)

#Significant difference between women drinkers and non-drinkers (Mann-Whitney U-test, p≤0.05)

CI=Confidence interval

HDL-C=High-density lipoprotein cholesterol

**Table 5.** Mean (SD) of gamma glutamyl transferase (GGT), percentage carbohydrate-deficient transferrin (%CDT), and percentages of elevated GGT and %CDT by reported alcohol consumption and gender

Gender	Alcohol consumption g/day^a^	N	GGT Mean (SD) (U/L)	95% CI	% with elevated GGT^b^	%CDT Mean (SD)	95% CI	% with elevated %CDT^c^
Men	0	236	91.0 (296.5)	53.6-128.4	18.9	2.9 (1.4)	2.7-3.1	47.7
	>0.01-15.99	177	129.7 (250.0)	93.2-166.1	39.3	3.5 (1.6)	3.3-3.8	66.8
	16.00-30.00	87	136.8 (150.5)	105.0-168.5	50.6	4.1 (1.7)	3.7-4.5	75.6
	≥30.01	150	188.7 (187.0)	158.8-218.5	64.1	4.1 (1.8)	3.8-4.3	81.3
Women	0	708	50.7 (65.1)	45.9-55.4	25.6	2.5 (1.0)	2.4-2.5	38.1
	>0.01-15.99	200	98.6 (133.6)	80.4-116.8	51.9	3.0 (1.3)	2.8-3.2	56.7
	16.00-30.00	57	136.0 (195.5)	85.4-186.5	66.7	2.8 (1.2)	2.4-3.1	55.2
	≥30.01	94	198.9 (271.1)	144.5-253.2	77.3	3.1 (1.3)	2.8-3.3	56.8

^a^Quantitative food frequency questionnaire method

^b^GGT: Men ≥80 U/L and women ≥50 U/L

^c^Percentage CDT: For both men and women ≥2.6%

CI=Confidence interval

N=Number of subjects

The reliability of reported alcohol consumption in all studies, including this one, remains debatable, due to inherent measurement error in QFFQs, specifically in quantifying intakes, imprecise portion-size assessment (volume consumed), and cognitive difficulty of completion. In this study, the QFFQ and the 24-hour recalls were done at the study site because many of them could not complete this on their own at home due to lack of understanding. In the QFFQ, respondents tend to give the modal rather than the arithmetic volume of consumption and to underreport events with an unusually high alcohol-use. African men, habitually drink alcohol through sharing large mugs or ‘quotes’ of traditional brewed alcohol, making it difficult to recall and quantify individual intakes after consuming in groups. In the current study, if sharing of mugs or quotes was done, we assumed that everyone sharing consumed the same amount.

Guidelines from developed countries (Europe, UK, and North America) recommend that daily alcohol consumption should not exceed 5% of total energy intake, or approximately 20 g for men and 15 g for women (25,26). Both men and women drinkers in this population reported high mean intakes of 29.9 and 23.3 g/day respectively, which is far greater than recommended. High standard deviations (30.0 and 29.1) for both genders illustrate a wide range in intakes in these groups. Almost two-thirds of the men in this sample reported to be drinkers compared to only a third of the women. This study was conducted when there were no festivals or celebrations. Due to urbanization, this population is at present experiencing a health and dietary transition (1). A cross-sectional, comparative, population-based study—the Transition and Health during Urbanization of South Africans—conducted between 1996 and 1998 in the same localities (North-West Province) showed mean self-reported intakes for men and women drinkers of 30.2 and 11.4 g/day respectively (27). Our results illustrate that mean alcohol intake among women has doubled (23.3 g/day) since then, suggesting women have either increased their consumption or are more honest in reporting their intakes. This may illustrate a reduced stigma surrounding alcohol consumption by women. Male drinkers in both studies (29.9 vs 30.2 g/day) had high intakes but did not differ, suggesting that their drinking patterns remained unchanged and/or their reporting maybe more honest and accurate. Both self-reported men and women drinkers had significantly higher levels of GGT and %CDT than self-reported non-drinkers. If the recommended cutoff values are used (GGT: men ≥80 U/L and women ≥50 U/L, %CDT: ≥2.6% for both men and women), both men and women drinkers had extremely high GGT and %CDT values, indicating a chronic drinking pattern for both genders. When discussing %CDT *per se*, it should be noted that other factors besides chronic intake of alcohol are known to be responsible for high %CDT values. Inborn errors due to rare genetic D-variants of transferrin (28) in glycoprotein metabolism (29) and liver diseases, such as cirrhosis, primary biliary cirrhosis, chronic active hepatic and chronic viral hepatitis (30–33), may cause false-positive results. These conditions cannot be totally ignored for this population but the genetic conditions are rare, and all participants who had a diagnosed chronic condition were excluded from this study.

Chronic iron deficiency and pregnancy may also influence the response of CDT (absolute measure) to heavy alcohol consumption (34,35), although this has been thought not to influence %CDT (the relative measure) (21). Although the apparently healthy non-pregnant population was included in this study, the possible contribution of iron deficiency should also be considered as iron deficiency is a major public-health problem in South Africa. Non-drinker men also showed mean elevated levels of %CDT, additionally suggesting the possibility of misreporting. Elevated mean GGT values in this sample suggest high alcohol consumption for both male and female drinkers. Non-drinker men similarly had an elevated mean GGT value of 91 U/L. However, elevated GGT values are also associated with other conditions, such as obesity, diabetes mellitus, hepatobiliary disorders, smoking, and CVD (13). The high GGT levels among the drinkers could, in part, be explained by smoking, since both drinker men and women significantly smoked more than their non-drinker counterparts. Although smoking and BMI were statistically adjusted for, the issue of residual confounding should be taken into account.

Both %CDT and GGT showed a relatively and highly significant correlation with self-reported alcohol intakes, confirming that increased levels of %CDT and GGT values can reflect high alcohol consumption. GGT showed better correlations with self-reported intakes than %CDT. The observed strong significance in these correlations could be, in part, mainly due to the large sample-size of this study. However, correlations between the two biological markers were low, suggesting that the responses of %CDT and GGT to alcohol consumption may occur via different mechanisms (36). Elevation of GGT in serum probably reflects its enhanced hepatic synthesis rate, increased transport to the liver plasma membranes, and liver injury (37). The mechanisms responsible for the increase in serum CDT levels are still being investigated. One possibility is that alcohol consumption decreases the activity of glycoprotein glycosyltransferase enzymes, namely sialyltransferase, galactosyltransferase, and N-acetylglucosamine transferase found predominately in hepatic Golgi complexes (38). These are primarily responsible for addition of sialic acid and other carbohydrate moieties to the transferrin polypeptide chain via a process known as glycosylation (39). Alcohol consumption has also been thought to increase the activity of sialidase that is involved in the removal of carbohydrate moieties from transferrin (38). The correlations between the two biomarkers and reported intakes are comparable with data from Russian settings where extensive work pertaining to these associations has been done (13). The combined use of CDT and GGT has been recommended as a better tool for identifying alcohol consumption and risky drinking patterns (14,40). As previously mentioned, %CDT has been shown to be a better marker than CDT as it eliminates variations in transferrin.

Comparing the two dietary methods, the QFFQ showed stronger correlations with the biological markers than the 24-hour recall. A highly significant correlation was observed between the two different sets of questionnaire assessing alcohol consumption. After controlling for smoking and body mass index, all correlation coefficients between biomarkers and reported intakes decreased, illustrating a negative directional effect of the two confounders. Whether %CDT and GGT are suitable as proxy markers of alcohol intake in African populations remains unclear. This study observes that, in this population, the levels of both GGT and %CDT for the self-reported non-drinkers were higher than the considered normal ranges based on studies done on other populations. Therefore, it seems reasonable to conclude that both GGT and %CDT could possibly misclassify subjects as drinkers or there is possibly a high misreporting rate for alcohol intakes among participants claiming to be abstainers. However, GGT and %CDT levels increased progressively with increased intakes of reported alcohol consumption.

### Conclusions

The findings of the present study highlight the importance of better-clarifying the relationship between reported alcohol intakes and known biomarkers, in populations in which the specificity and sensitivity of these markers are unclear. An association was observed between reported alcohol intakes and selected biomarkers (%CDT and GGT). It should be noted that alcohol consumption, especially in African settings, may not be reliably estimated by self-reporting and that consumption is relatively high. It seems necessary to revise and set new cutoff values (biomarkers) for African populations, through robust dose-response studies. However, the dual use of self-reported alcohol intakes and biological markers are both important in identifying and assessing risky drinking patterns.

## ACKNOWLEDGEMENTS

This project/work has been supported, in part, by South Africa-Netherlands Research Programme on Alternatives in Development (SANPAD); South African National Research Foundation (NRF GUN numbers 2069139 and FA2006040700010); the North-West University, Potchefstroom, South Africa; and the Population Health Research Institute, ON, Canada. The authors express their thanks to the PURE-South African team (including the research team, fieldworkers, and office staff in the Africa Unit for Transdisciplinary Health Research, Faculty of Health Sciences, North-West University, Potchefstroom, South Africa) and PURE International: Dr. S. Yusuf and the PURE project office staff at the Population Health Research Institute, Hamilton Health Sciences and McMaster University. ON, Canada.
